# Managing outbreaks of highly contagious diseases in prisons: a systematic review

**DOI:** 10.1136/bmjgh-2020-003201

**Published:** 2020-11-16

**Authors:** Gabrielle Beaudry, Shaoling Zhong, Daniel Whiting, Babak Javid, John Frater, Seena Fazel

**Affiliations:** 1 Department of Psychiatry, University of Oxford, Oxford, Oxfordshire, UK; 2 Department of Psychiatry, The Second Xiangya Hospital, Central South University, Changsha, Hunan, China; 3 Division of Experimental Medicine, University of California San Francisco, San Francisco, California, USA; 4 Peter Medawar Building for Pathogen Research, Nuffield Department of Medicine, University of Oxford, Oxford, Oxfordshire, UK

**Keywords:** systematic review, public health, avian influenza, prevention strategies, infections, diseases, disorders, injuries, COVID-19, prisons

## Abstract

**Background:**

There are reports of outbreaks of COVID-19 in prisons in many countries. Responses to date have been highly variable and it is not clear whether public health guidance has been informed by the best available evidence. We conducted a systematic review to synthesise the evidence on outbreaks of highly contagious diseases in prison.

**Methods:**

We searched seven electronic databases for peer-reviewed articles and official reports published between 1 January 2000 and 28 July 2020. We included quantitative primary research that reported an outbreak of a given contagious disease in a correctional facility and examined the effects of interventions. We excluded studies that did not provide detail on interventions. We synthesised common themes using the Synthesis Without Meta-analysis (SWiM) guideline, identified gaps in the literature and critically appraised the effectiveness of various containment approaches.

**Results:**

We identified 28 relevant studies. Investigations were all based in high-income countries and documented outbreaks of tuberculosis, influenza (types A and B), varicella, measles, mumps, adenovirus and COVID-19. Several themes were common to these reports, including the public health implications of infectious disease outbreaks in prison, and the role of interagency collaboration, health communication, screening for contagious diseases, restriction, isolation and quarantine, contact tracing, immunisation programmes, epidemiological surveillance and prison-specific guidelines in addressing any outbreaks.

**Discussion:**

Prisons are high-risk settings for the transmission of contagious diseases and there are considerable challenges in managing outbreaks in them. A public health approach to managing COVID-19 in prisons is required.

**PROSPERO registration number:**

CRD42020178827

**Key questions**

**What is already known?**

- Prisons and other custodial facilities are high-risk environments for infectious disease outbreaks such as COVID-19.
- People in prison (PIP) may be susceptible to serious complications of COVID-19 due to increased prevalence of underlying health conditions.
- Prisons are porous environments, and thereby prison outbreaks have the potential to spread to surrounding communities.

**What are the new findings?**

- Screening, contact tracing and isolation appear to be the most applicable infection control strategies.
- Symptom screening can be ineffective as PIP may hide symptoms due to stigma, lack of trust in medical confidentiality in prisons, and to avoid prolonged medical isolation.
- Effective prison population reduction strategies, such as releasing persons at low risk of future serious offending and not incarcerating those charged with low-level offences, represent a major research gap.

**What do the new findings imply?**

- PIP and correctional staff should be communicated clear and up-to-date information about health risks, and prevention and control measures being implemented.
- Extensive collaboration between prisons and public health authorities is essential to leading a comprehensive public health response that takes into account the particular environmental and physical conditions, healthcare services and security constraints of each prison.
- The benefits of prolonged infection control strategies need to be weighed against the potential negative consequences of such measures on the mental health of PIP.

## Introduction

COVID-19 has developed into an international public health crisis accompanied by restrictions on daily life and more targeted measures (eg, travel bans, school closures and remote working). In prisons, barriers to translating such interventions are considerable, and there is a high risk of rapid transmission due to high population density and turnover, overcrowding and frequent movements within and between establishments.^1 2^ In addition, there is a high proportion of people in prison (PIP) who may be more vulnerable to severe COVID-19 due to underlying risk factors (such as male sex, older adults and having chronic underlying health conditions).^3–5^ In England and Wales, for example, around 95% of PIP are men, and 4% are aged 60 and older.^6^ Moreover, available US data suggests that Black, Latinx and Indigenous communities, which are over-represented in the criminal justice system,^7 8^ are disproportionately affected by COVID-19. Pre-existing structural determinants of health including systemic racial/ethnic and socioeconomic inequalities (eg, working in high-risk occupations, lack of access to healthcare, higher rates of public transport use, living in multihousehold accommodation) have been exacerbated by the pandemic. Structural inequalities may therefore contribute to the disproportionate incidence of COVID-19, associated severe illness and mortality in these communities.^9–14^ Finally, challenges to control infections may be compounded by poor prison medical services in some countries and the prioritisation of security over health needs.^15–17^

There is some evidence of high rates of infection of COVID-19 in prison. One non-governmental organisation estimated that in early June 2020, across 79 countries, 73 254 PIP had tested positive for COVID-19, of which more than 1100 had died from complications.^18^ Overall, infection rates in custodial facilities both among PIP and staff appear to be higher than in the general population, including in the USA,^19^ and England and Wales,^20^ although it is not known whether these are driven by differential testing. Some clusters have also been reported, including in one prison in Michigan, USA, where COVID-19 rates were over 10% in PIP and 20% in staff.^21^ Nevertheless, with prison populations worldwide amounting to around 10.7 million,^22^ and more than 30 million people circulating through prison every year,^23^ some jurisdictions have included prisons as part of the public health approach to dealing with this pandemic. Some have issued guidance for prisons and detention centres on how to manage COVID-19,^24^ and some have released PIP, partly due to legal challenges.^25^ Additional restrictions such as stopping visits to prison have been added. These and other measures have resulted in disturbances, including riots in prisons in many countries. In March 2020, riots in Italy led to the death of 12 PIP.^26^ In Colombia, another led to the death of 23 PIP and 83 injuries,^27^ and in Venezuela, a riot left 40 people dead and 50 injured.^28^

A number of commentaries and surveys in specific geographical areas have been published about the implications of COVID-19 in prison,^2 25 29–35^ but these lack any systematic evaluation of the efforts to mitigate the effects of highly contagious diseases in prison. Therefore, we have undertaken a systematic review of evidence on the management of outbreaks of highly contagious diseases specific to prisons to inform public health responses to COVID-19. We chose not to focus on bloodborne diseases such as HIV, hepatitis B and hepatitis C virus, as their mode of transmission is not directly relevant to airborne viruses such as COVID-19. The generalisability of this review’s findings to other custodial settings, such as police and detention centres, will be discussed.

## Methods

We conducted a systematic review of outbreaks of contagious diseases in correctional facilities according to the Preferred Reporting Items for Systematic Reviews and Meta-Analyses and the Synthesis Without Meta-analysis guidelines.^36 37^ We identified quantitative studies of primary research and the following electronic databases were searched from 1 January 2000 to 28 July 2020: Medline, Embase, PsycINFO, Global Health, Cochrane Database of Systematic Reviews, Cochrane Central Register of Controlled Trials, Web of Science Core Collection, and National Criminal Justice Reference Service. Our search strategy featured a combination of search terms relating to both PIP (ie, prison*, incarcerat*, custod*, imprison*) and outbreaks (ie, outbreak*, transmission, epidemic*, pandemic*). The detailed search strategy can be found in online supplemental appendix A.

10.1136/bmjgh-2020-003201.supp1 Supplementary data

Citations were screened independently by two researchers (GB and SZ). Studies were eligible for inclusion if they were published after 2000, reported a suspected or confirmed outbreak of a given contagious disease, as defined by authors, in a correctional facility and examined intervention effects. Correctional facilities were defined as those housing people in custody (eg, police custody suites, detention centres, jails and prisons). We solely included quantitative studies of primary research that were written in English and were published as either peer-reviewed journal articles or official governmental reports. Studies using different research designs to provide an empirical evaluation of diverse intervention effects were considered.^38^ As a result, qualitative studies, systematic reviews, commentaries, and epidemiological studies that did not investigate a given outbreak or examine intervention effects (eg, prevalence or case–control studies) were excluded. For instance, research reporting on high-incidence contagious diseases and targeted interventions taken to control their spread were not included without mention of a specific outbreak.^39 40^ All full-text articles were reviewed by two reviewers (GB and SZ). Any discrepancies were resolved through discussion with a third author (SF). No structured formal quality assessment was used in the review process, but inclusion was limited to peer-reviewed articles and governmental reports, and papers with key background characteristics and information on the disease outbreak. All eligible papers were included in the qualitative analysis.

We developed a standard data extraction form to collect information from eligible studies on year of publication, geographical location, outbreak setting, population, type of contagious disease, outbreak period, number of confirmed cases, and detailed information regarding contaminated individuals and deaths, if applicable. Three reviewers (GB, SZ and DW) conducted qualitative data analyses by identifying common themes across the included studies. GB and SZ read and extracted findings from all included studies, with a particular focus on (1) presentation of the outbreak; (2) description of outbreak control measures; (3) their impact on PIP, staff and the local community. The two reviewers independently identified common themes in the included studies using a thematic analysis approach, and subsequently compared their analyses. Overlapping themes were combined and unique ones were retained (including specific recommendations). Discrepancies were resolved through discussions with another reviewer (DW). Finally, we organised the themes to provide an overall interpretation of the findings, and referenced each element with the study from which it was derived. This narrative synthesis was performed using NVivo V.12 Software for qualitative data analysis.^41^

People-centred language is employed to describe people who experience incarceration (ie, ‘PIP’ in place of ‘prisoners’). This terminology refers to incarcerated persons only and does not include members of staff.^42^

### Patient and public involvement

This research was done without patient or public involvement.

## Results

### Study selection and characteristics

**Figure 1:**
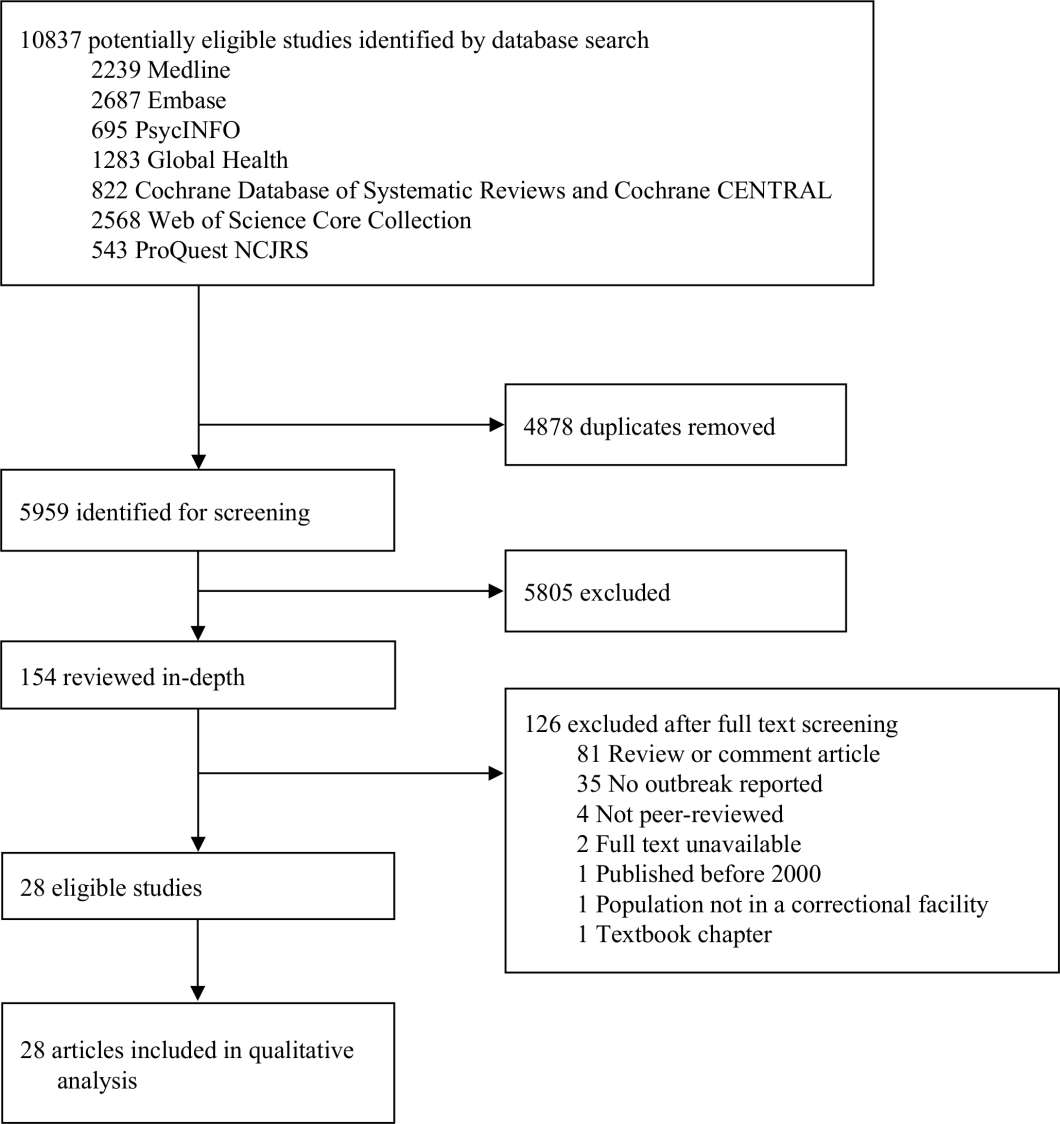
PRISMA Flowchart.PRISMA, Preferred Reporting Items for Systematic Reviews and Meta-Analyses.

The initial search yielded 5959 relevant articles, of which 154 full-text articles were screened. Twenty-eight publications met inclusion criteria and were included in the final sample (figure 1).^43–70^ Characteristics of the included studies are summarised in table 1. Eight studies described outbreaks of tuberculosis (TB),^45–47 54 56 59 62 63^ seven of influenza (types A, eg, H1N1 and H3N2, and type B),^43 44 48 49 53 64 68^ six of varicella,^52 57 58 60 61 65^ four of measles^50 51 55 66^ and one each of mumps,^67^ adenovirus type 14p1^69^ and COVID-19.^70^ The outbreaks occurred in seven different countries: 12 in the US^45–47 54 56 59 62 63 66 70^; six in Australia^43 50 53 58 64 68^; four in the UK^51 55 57 69^; three in Canada^44 60 67^ and one each in China (Taiwan),^49^ Italy^65^ and Switzerland.^52^ All described outbreaks occurred in adult custodial facilities, with the exception of one which took place in a youth custody facility.^60^ One outbreak was observed in a privately operated immigration detention centre,^66^ but the remainder were in jails and prisons. No studies reporting on police custody were identified.

**Table 1:** Study characteristics

Study	Geographical location	Outbreak setting	Population	Contagious disease	Outbreak period	Information regarding P1	Confirmed cases	Information regarding the cases	Fatalities	Service responsible for outbreak control
Awofeso (2001)^43^	Australia	Psychiatric ward within the correctional facility	17 PIP, 18 prison officers and 24 staff	Influenza type A (H3N2)	29 August–8 September 2000	P1 was infected during a visit from a member of the public	9 cases	6 PIP, 2 nursing staff, and one patient care assistant; Age (range 25–70 years); All of non-Aboriginal background	None	CHS
Besney (2017)^44^	Canada	Maximum security remand facility	Approximately 1200 PIP	Influenza type A (H1N1)	10–18 December 2013	P1 was transferred from another correctional facility that was experiencing increased influenza activity	6 cases	All males; Age (*M*=41, range 24–54 years); 3 Canadian-born non-Indigenous, 2 Canadian-born Indigenous and one foreign-born	None	A combination of CHS and CPH
Bur (2003)^45^	USA	Urban community and jail	344 PIP housed with P1	TB	April 2000– September 2001	P1 was diagnosed in the community; Home visit revealed that his house was used for trafficking illicit drugs, and was frequented by many people; He had been in jail from December 1999 through January 2000	18 cases	Age (*M*=39, range 1–66 years); Male (78%); African American (89%); HIV-seropositive (39%)	One deceased	A combination of CHS and CPH
Centers for Disease Control and Prevention (CDC) (2000)^46^	USA	State correctional facility housing HIV-infected PIP	323 PIP housed in the same dormitory as P1	TB	1999–2000	P1 (who was HIV-positive) was diagnosed at a community hospital	31 cases	All current or former PIP; All non-Hispanic Black men born in the USA and HIV-seropositive; Age (*M*=36, range 23–56 years)	None	A combination of CHS and CPH
CDC (2004)^47^	USA	Multiple correctional facilities	800 possible contacts, 318 of which were identified	TB	2002–2003	P1 visited a homeless shelter, 3 different jails and a state prison during the infectious period	2 cases	2 TB cases and 47 additional LTBI cases; Active cases were cellmates of P1	None	A combination of CHS and CPH
CDC (2012)^48^	USA	Two correctional facilities: one medium to maximum security prison and one minimum security prison	Facility A (916 PIP and 410 staff members); Facility B (222 PIP and 65 staff members)	Influenza type A (H1N1) and type B	March 2011	Not provided	Facility A: 6 cases Facility B: 1 case	Facility A (influenza A(five were H1N1pdm09 and one was unsubtypable), Age (*M*=37, range 24-57 years, only one had been vaccinated previously); faciliaty B (influenza B)	One deceased	A combination of CHS and CPH
Chao (2017)^49^	China (Taiwan)	Prison	2690 PIP	Influenza type A (H1N1)	February–April 2013	Not provided	5 cases	2 had HCV/HIV and one suffered from hypertension	None	A combination of CHS and CPH
Chatterji (2013)^50^	Australia	High-security correctional facility	Approximately 900 PIP and 450 staff	Measles	October–November 2013	Not provided	17 cases	14 PIP and three correctional centre staff; age (*M*=28, range 18–41 years); Sex (3 female and 14 male); Indigenous status (one Indigenous, 13 non-Indigenous and 3 unknown)	None	A combination of CHS and CPH
Crick (2014)^51^	UK	Adult prison (category C/D)	210 PIP	Measles	December 2012–February 2013	P1 was a member of the prison staff; had free access to all areas of the prison; continued to work throughout the prodromal period	8 cases	Three distinct waves of infection	None	CHS
Gétaz (2010)^52^	Switzerland	Pre-trial prison	540 PIP, 243 prison officers and 37 healthcare workers	Varicella	April 2009	P1 was a PID who sought medical attention 48 hours after the beginning of a rash characteristic of chickenpox	2 susceptible cases	Remained in a small cell with P1 and were considered as household contacts	None	CHS
Guthrie (2012)^53^	Australia	Custodial facility (all security levels)	Average daily occupancy in 2009 (163 PIP), in 2010 (214 PIP)	Influenza type A (H1N1)	2009–2011 (during influenza season: 1 July–30 September)	Not provided	2009: 1 case (also reported in Turner and Levy, 2010) 2010: 4 cases 2011: 2 cases	Not provided	None	CHS
Jones (2003)^54^	USA	Urban jail and surrounding community	Not provided	TB	1995–1997 (jail outbreak period); January 1998–August 1999 (follow-up period in surrounding community)	Not provided	Jail: 43 cases Community: 81 cases	6 of jail cases and 19 of community cases were infected with the jail outbreak strain	None	A combination of CHS and CPH
Junghans (2018)^55^	UK	Prison	More than 1500 PIP	Measles	July 2016	P1 was a member of the prison staff	8 cases	1 probable, 5 possible and 2 confirmed cases	None	A combination of CHS and CPH
Lambert (2008)^56^	USA	Privately managed medium-security state facility	Daily census of approximately 2000 PIP	TB	2003–2004	Not provided	7 cases	All males; age (*M*=34, range 24–58 years); 1 HIV-seropositive; four reported drug or alcohol abuse	None	A combination of CHS and CPH
Leung (2014)^57^	UK	State prison	Approximately 2000 staff and 5000 PIP	Varicella	16–23 January 2010 and 12 February–25 March 2011 (rash onsets)	P1 worked in the kitchen	11 cases	Age (*M*=37, range 19–58 years); None were immunocompromised or had any varicella-related complications; nine cases associated with the 2 outbreaks and 2 sporadic cases	None	A combination of CHS and CPH
Levy (2003)^58^	Australia	Four prisons, one prison hospital, the prison transport system, one courthouse	Over 300 PIP exposed to varicella during the outbreak	Varicella	4 weeks (exact dates not reported)	P1 was infected during a family visiting session	6 cases	5 confirmed cases and 1 probable case; 3 cases in prison A, 2 cases were infected in court transport van and 1 case at the prison hospital	None	A combination of CHS and CPH
Mohle-Boetani (2002)^59^	USA	Correctional-facility housing unit for PIP infected with HIV	More than 3200 PIP; 472 residing in the HIV unit	TB	1995	Not provided	15 cases	Age (*M*=35, range 30–43 years)	Two deceased	A combination of CHS and CPH
Moreau (2016)^60^	Canada	Youth custody facility	Approximately 280 youths, 14 health services staff and 85 security staff	Varicella	July 2013	P1 was a male youth	2 cases	All males; age (*M*=14, range 13–18 years); all cases and contacts were Canadian born	None	A combination of CHS and CPH
Murphy (2018)^61^	UK	Three correctional facilities	Not provided	Varicella	May 2016–January 2017	Prison A: P1 was a PIP who had herpes zoster Prison B: P1 was a PIP with ocular herpes zoster (not recognised as disseminated herpes zoster at that time) Prison C: P1 was a PIP who had herpes zoster lesions	Prison A: 5 cases Prison B: 3 cases Prison C: 1 case	Age (*M*=40, range 29–49 years); None were immunocompromised; one had prior varicella infection	None	A combination of CHS and CPH
Njuguna (2020)^70^	USA	Correctional and detention facility	Approximately 700 PID	COVID-19	April–May 2020	P1 was a staff member who reported symptoms of COVID-19 and later tested positive for SARS-CoV-2	110 cases	First 39 cases identified through active monitoring (29 March–7 May); Additional 71 cases identified through an investigation led by the Louisiana Department of Health and CDC (7–21 May)	None	A combination of CHS and CPH
Parcell (2014)^69^	UK	Two male-only prisons	Not provided	Adenovirus 14p1	1 Jan 2011–25 July 2011	Not provided	15 cases (13 confirmed and 2 possible)	Of the 13 confirmed cases: 7 PIP, 1 staff and 5 from non-prison communities; All of white Scottish origin; Age (range 23–70 years); Male (76.9%)	Three deceased	A combination of CHS and CPH
Saunders (2001)^62^	USA	Federal prisons	25 707 PIP	TB	January 1997–June 1999	Not provided	75 cases	46 cases (61%) were foreign-born; 15 (20%) were HIV-positive	None	CHS
Saunders (2001)^62^	USA	Detention centre	1830 PIP	TB	June 1997–December 1998	P1 was a PID	7 cases	Not provided	None	CHS
Sosa (2008)^63^	USA	State-run jail and prison	Not provided	TB	May 2005–May 2006	P1 was a US-born, HIV-negative PIP whose TST was negative	2 cases	US born; Age (range 20–29 years)	None	A combination of CHS and CPH
Turner (2010)^64^	Australia	Adult custodial facility	140 PIP	Influenza type A (H1N1)	July 2009	P1 was a 46 year old male PID and was infected during a family visiting session	1 case	Age (*M*=36 years); Remand status	None	A combination of CHS and CPH
Valdarchi (2009)^65^	Italy	Prison for women	424 PIP	Varicella	13 April–2 May 2005	P1 was a 26 year old HIV-positive Italian woman	5 cases	3 Italians and 2 Nigerians; two were HIV-positive; all were hospitalised on the end of the onset of symptoms	One deceased	CHS
Venkat (2019)^66^	USA	Privately operated detention facility housing US Immigration and Customs Enforcement PID	1425 PID and 510 staff members	Measles	25 May–8 August 2016	P1 was a PID who was hospitalised with symptoms	32 cases	23 PID and nine staff; Age (*M*=36, range 19–52 years); male (84%); three hospitalisations	None	A combination of CHS and CPH
Walkty (2011)^67^	Canada	Two prisons (one medium security)	135 PIP and 187 staff	Mumps	12 January–5 February 2009	P1 was a 28 year old PIP with clinical symptoms who was transferred from Prison B to Prison A	Prison A: 5 cases Prison B: 4 cases	Age (range 28–34 years); all males of self-identified Aboriginal ethnicity	None	CHS
Young (2004)^68^	Australia	Five prisons (Two maximum, one medium and two minimum; Three metropolitan and two rural)	Approximately 7900 PIP	Influenza type A (H3N2)	January 2003	Not provided	37 cases	35 PIP, one healthcare staff and one custodial officer; First 20 cases shared the same unit; Another 8 cases were from the same prison	None	A combination of CHS and CPH

CHS, correctional healthcare services; CPH, community public health; HCV, hepatitis C virus; LTBI, latent tuberculosis infection; PID, people in detention; PIP, people in prison; TB, tuberculosis; TST, tuberculin skin test.

Twenty reports were based on single-centre studies that gave a chronological description of an outbreak and the measures employed by the institution. Some also reported on multiple custodial facilities.^48 58 61–63 67 68^ The effectiveness of interventions could not be evaluated as the included studies were not designed to test them. However, one study considered expected secondary attack rates in an attempt to illustrate the effectiveness of implemented measures.^52^ We identified common themes from the detailed qualitative analysis with a focus on relevant observations, specific approaches, challenges encountered and recommendations for future outbreaks of contagious diseases (table 2).

**Table 2:** Summary of recommendations for managing infectious outbreaks in prison

Recommendation	TB	Influenza	Measles, mumps, varicella	Adenovirus	COVID-19 (hypothetical impact)
Interagency collaboration	++	*	*	*	++
Health communication	++	*	*	*	++
Screening for contagious diseases					
Symptoms	+	+	–	+	+ (Marginal)
Diagnostic	+	+	+	*	+
Immune status	–	–	++	–	Unclear
Restrictions, isolation and quarantine	++	+	++	+	++
Contact tracing	++	–	+	+	++
Immunisation programmes	–	+	++	–	–
Epidemiological surveillance	++	++	++	–	++
Prison-specific guidelines	+	+	+	+	+
Appropriate treatment	++	+/-	–	–	–

-No current potential impact; +limited impact; ++likely impact; *data inadequate to formulate robust recommendation. All recommendations with the exception of the COVID-19 ones are based on the literature identified from the review. COVID-19 recommendations stem from consensus based on considering general literature on COVID-19 and one included study.^70^ BJ and JF reviewed this literature, considered its applicability, and formulated the recommendations jointly.

TB, tuberculosis.

70

### Public health implications

Twelve studies underscored the potential community impact of prisons during an outbreak.^44 45 48 54 56 58 62–64 68–70^ In one TB study, over two-thirds of PIP identified as having been exposed had already been released,^45^ and in another US study, 23% of community TB cases were from a strain indistinguishable from a previous jail outbreak strain, most with no recent history of incarceration.^54^ In low security institutions, PIP can have extensive community contact,^51^ and additionally through court appearances, transfers and associated transport, prisons can have a significant impact on broader community transmission.^44 70^ One account of a TB outbreak identified that the index case had been in three different local prisons and one state prison while symptomatic, resulting in approximately 800 contacts.^47^ Other studies^54 58^ have found that correctional institutions can serve as important reservoirs of disease, and should be proactively monitored for emerging outbreaks.^68^ Transmission from community to prison can also occur through admissions of persons who are newly detained,^71^ community visitors from high-risk settings^43^ and staff members.^51 55 57^ Stigma against PIP and limited appreciation of the permeability of prisons to surrounding communities can act as potential obstacles to their inclusion in the public health infrastructure.^56 62 63 70^

### Interagency collaboration

Interagency collaboration is integral to managing infectious disease outbreaks.^44 50 56 60 63 64 66–70^ Studies described early establishment of a designated interagency group to coordinate the response,^60^ involving prison staff, the local state health department, public health laboratory, public health unit and hospital services.^50^ In two studies, prison authorities notified other correctional facilities to prevent spread through transfers.^66 67^ Links with the local public health department also assisted contact investigation for those who had been released.^56^ Even well-staffed health facilities within prisons can rapidly become overwhelmed during an outbreak, and requesting help from public health officials should occur at an early stage.^59^

### Health communication

Prisons have limited access to external information (and mostly no internet provision within cells), so special measures are required to convey public health messages.^72^ Twelve investigations described approaches to this.^43 44 46 50 56 57 59 60 63 66 67 69^ Initiatives targeted PIP (current and recently released),^59^ staff,^43 46 56 57 63^ both these groups,^44 60 66^ visitors and the general public.^50 67^ Formats included written materials,^43 66^ individual and group clinics,^59^ debriefing sessions,^60^ press releases and use of social media.^66^ In four studies, key messages were disseminated with limited details,^44 50 56 57^ whereas in one report of a TB outbreak in individuals with HIV more intensive engagement was used.^59^ In others, content included disease-specific information (eg, transmission, symptoms, complications), infection control measures (eg, respiratory etiquette, hand hygiene practices, sharing of food or drinks), and the importance of adherence with treatment or immunisation. Two studies used culturally sensitive tools available in different languages.^52 66^

### Screening for contagious diseases

Fourteen reports suggested that screening was essential to mitigate the impact of the outbreak.^45–48 56–59 62 63 66 68–70^ Some recommended selective screening, which focused exclusively on symptomatic^69^ or potentially exposed PIP and staff,^45 46 66 70^ with one study using at home testing for symptomatic staff,^43^ while others proposed a universal approach,^48 56–58 62^ including extending screening to staff family members.^43^ Two studies^56 70^ recommended that screening be conducted on multiple occasions (eg, following exposure, on entry (for PIP) or employment (for staff), and on an annual basis),^56^ and another reported daily temperature and symptom monitoring in affected prison units.^66^

Both symptom screening (ie, temperature screening and oxygen saturation measurements) and serial testing were used to mitigate transmission of SARS-CoV-2 infection in the one COVID-19 study. Persons in affected dormitories were tested on three separate occasions during the investigation period in order to classify cases as asymptomatic, presymptomatic, or symptomatic.^70^ This dual strategy increases identification of presymptomatic or asymptomatic persons infected with SARS-CoV-2 who could potentially be missed if symptom screening alone was employed.^73^ Moreover, symptom screening can also be ineffective due to related disincentives associated with reporting illness among PIP such as avoidance of medical isolation, and, in some cases, costs incurred by medical care.^74^

All studies on varicella, measles and mumps reported information in regards to serology screening to detect susceptibility and identify at-risk individuals.^50 52 57 58 60 61 65^ Screening for measles immunity on prison entry was common practice,^50 51 55 66^ with the exception of one study of prisons in Queensland.^50^ For varicella, a variety of approaches were adopted in terms of targeting serological testing; in a Swiss study, testing was offered to all PIP contacts of the index case regardless of whether there was history of varicella, and staff members with no prior history of varicella^52^; in a US prison it was offered to exposed individuals and those born outside the US or in the US after 1979;^57^ and in an Australian prison system outbreak serological tests were offered to all HIV antibody positive PIP and contacts who had an undetermined past history of varicella.^58^ Another US investigation recommended serological testing for exposed PIP who lack clear documentary evidence of immunisation or prior clinical diagnosis.^61^

### Restrictions, isolation and quarantine

Restrictions on movement and mixing between PIP can be challenging, with logistical implications for essential legal and prison processes, as well as potential effects on rehabilitation and well-being because of disruption to structured therapeutic programmes, social contacts and legal advice. The range of restrictions varied, including limiting movements within a prison,^43 44 50 53 57 60 68^ transfers in and out,^43 48 50 51 55 66 67^ stopping new entrants,^44 48 60 66^ transport of affected individuals,^44^ family and legal visits,^50 66 68^ school programmes,^60^ and transport between prisons and courts.^57 66^ Some studies monitored those who had been transferred to other facilities.^50 67 67^ Few studies specified the definition of restricted movements. However, one described restrictions to education, prison employment, religious activities and outdoor exercise.^57^ One study highlighted how they safely maintained visits during a H1N1 pandemic; however the institution was relatively new, accommodated half of PIP in self-contained cottages, and was not yet at capacity, and so was able to rapidly instigate isolation and quarantine.^53^

Isolating PIP, either due to suspected or confirmed disease or for quarantining potential contacts,^52 53 57 58 66 70^ may be effective but can be logistically difficult, as well as of poor acceptability to PIP given its more typical role as a form of punishment in this setting.^51^ Due to differences in available space, where described in included studies suspected and confirmed cases were placed in local hospitals,^69^ prison healthcare facilities,^44 52 57 58 61 70^ airborne-infection isolation (AII) rooms,^47 57 59 63^ their own cells^51 58 60 64 67 68^ and restricted units.^66 70^ Negative pressure rooms were used when feasible.^61 62^ In the COVID-19 study, test results were used to inform whether PIP should remain in quarantined dormitories or be transferred to another facility for medical isolation.^70^ One study reported the exclusion of symptomatic staff,^53^ and another reported isolation or exclusion from work (during the incubation period) of PIP and staff who refused vaccination programmes.^50^

### Contact tracing

Contact investigation to identify potential asymptomatic cases is a key component of outbreak response, especially where there is prevalent nosocomial transmission (as is the case with COVID-19) (see table 3 for a summary of transmission routes by infection). Eleven studies reported approaches to this in prison.^45–47 51 56 58 59 66 68–70^ Methods included case interviews,^58 59 68^ a prisoner tracking system,^56 59^ reviewing case movements within the prison,^69^ and communication by mail.^56 59^ Their scope differed considerably across studies. Some were limited to in-prison contacts,^46 51 58 68 70^ while others also included recently released or transferred PIP and community contacts.^45 56 59 66^ One study identified potential exposure in the community through staff index cases at multiple locations (eg, food shops, healthcare facilities, petrol stations, etc).^66^ Letters were sent to released people with known contacts to inform them of possible exposure and the need for screening.^56 59^ Individual contact tracing can be unfeasible given the potentially large numbers.^50^ In a measles outbreak in one English prison (with 210 PIP), the index case was a staff member and the whole prison population was considered as potential contacts during the prodromal period due to extensive mixing.^51^ A concentric circle approach was proposed for investigations in the surrounding community in another study,^45^ where efforts first focus on the closest contacts and then extend to those with less exposure if the infection rate surpasses the population norm.^75 76^ Such approach is beyond the scope of individual prisons and thereby requires collaboration with the local public health agency. The importance of accessing re-arrest data to trace individuals who may have been detained in multiple facilities over short periods was also noted.^45^

**Table 3:** Transmission route and clinical characteristics of highly contagious diseases

Disease	TB	Influenza	Measles, mumps and varicella	Adenovirus	COVID-19
Transmission routes	Airborne, ?prolonged contact	Droplet, fomite ? airborne	Airborne (measles, varicella), droplets (mumps)	Droplet, fomite	Droplet, ?fomite ?airborne
Presymptom transmission	Unclear but less likely	Yes, 12 hours before symptom onset, but less contagious	Yes (during prodrome but before rash for measles, yes (varicella 1–2 days before rash), yes mumps (2 days before parotitis)	Unknown but unlikely	Yes
Incubation period	Weeks months	1–4 days	12–25 days mumps, 10–12 days measles, 10–20 days varicella	2–14 days	1–14 days (median 4–6 days)
Isolation period	Until appropriate treatment started	Not known	Varicella: until rash ‘crusted’; measles: until 4 days after rash onset; mumps: ‘from several days before the parotid swelling to several days after it appears’ (PHE)	Not known	7 days for mild/moderate disease and 14 days for severe disease

?=Uncertain as robust conclusions cannot be formulated from current data.

PHE, Public Health England; TB, tuberculosis.

### Immunisation programmes

Twelve studies described postexposure immunisation programmes where applicable.^43 44 48 50–52 55 57 60 65–67^ Studies reported on a mass immunisation approach that included PIP^52 55^ and both PIP and staff.^50 51 57 65–67^ In one study of an influenza outbreak, the immunisation strategy included uninfected PIP residing in proximity to affected units.^44^ A second study reported a similar strategy extended to prison officers and medical staff.^43^ Public health nurses provided assistance by offering immunisation to PIP and prison staff presenting with influenza-like illness in one study.^48^ Other studies targeted immunisations based on evidence of immunity status in varicella and measles.^55 60^ Vaccine shortages were reported for influenza (ie, H1N1 and H3N2),^64 68^ One solution was the targeted use of an antiviral drug (oseltamivir) in exposed persons.^64^

### Epidemiological surveillance

Sixteen studies found that careful information recording was critical to effective outbreak management.^45 46 48 49 51–53 55–57 60 62 64 66 69 70^ Recommended initiatives included PIP,^45 60^ visitor,^64^ and staff movement registries,^56^ and medical records that feature symptom data, infectious disease and immunisation status.^46 48 51 55 62 66 70^ Two studies reported that rapid access to immunisation status could improve the effectiveness of any response given and that self-reported history of disease should be considered unreliable.^52 58^

### Prison-specific guidelines

Ten studies reported that there were no readily available correctional guidelines for managing epidemics at the time of the outbreak.^45 47 48 50 51 53 56 60 63 66^ One study found that prison authorities relied on collaborative guidelines that had been developed prior to the outbreak.^44^ Other sources of information included prison-based guidelines, guidelines for the general public,^57 62^ expert advice,^50^ general guidelines from the US Centers for Disease Control and Prevention,^45 51 53 56 66^ and the UK Health Protection Agency^69^ and interim guidance.^53^ One study reported new guidelines being introduced as a result of an outbreak, and recommended surveillance and tracing of community contacts.^59^

## Discussion

This systematic review on highly contagious infections within prisons identified 28 studies from seven countries. Nine themes were identified, including public health impacts, interagency collaboration, health communication, screening for contagious diseases, restrictions, isolation and quarantine, contact tracing, immunisation programmes, epidemiological surveillance and prison-specific guidelines. Although the heterogeneous research designs of included studies did not allow for quantitative comparison of responses described, we identified consistent themes in relation of the importance of immunisation (if possible), screening new entrants, contact tracing and isolation of suspected cases. Some unique challenges to prisons were also described, including high rates of movement between and within establishments, and the large number of potential contacts based on the high turnover in many prisons, regular visitors and regular association with prison staff. These may be exacerbated in certain settings, such as local jails and immigration removal centres, where there can be very high turnover and overcrowding is common with poorer physical infrastructure. Epidemiological surveillance is therefore more important in these settings.

PIP may be more likely to hide symptoms due to stigma, lack of trust in medical confidentiality in prisons, and the implications of further restrictions in people whose liberty has already been deprived. At the same time, COVID-19 is quite different to the outbreaks described in previous work, which have been all been self-limiting. It could spread widely and quickly within an institution in the absence of the right interventions (eg, the Diamond Princess cruise ship outbreak).^77^ As such, there could be a shift in balance of the direction of travel of the infection from it mostly entering a prison to prisons becoming a reservoir for community infection.^78 79^ Thus, the most applicable evidence is in relation to screening and contact tracing with appropriate isolation procedures.^70^ Part of this strategy will require a particular emphasis on staff health and safety to implement testing, tracing and isolation of suspected and confirmed cases of PIP. In terms of contact tracing, the use of tracking apps needs further consideration, but such an approach is unlikely to be easily implemented as PIP do not typically have access to mobile phones or other personal internet-enabled devices. Moreover, tracking previously detained persons on release would be impractical because many such persons will not have phones or will have new ones on release, and it will also raise other potentially complex human rights and legal considerations. The feasibility and acceptability of such approach would also be dependent on the locality and region with particular criminal justice approaches, capacities and the level of integration with public health and primary care. One concern might be that information on geographical location is used by police for future investigations even if the initial consent was for health use. If the confidentiality of the app is not ensured and trusted by people leaving prison, then the uptake of such tracking apps will likely be low.

Although we did not formally assess study quality, all included investigations were observational and reported on a specific outbreak, subsequent prison responses and their impact. The literature was confined to either highly contagious diseases with low morbidity and mortality (measles and varicella), moderate contagious outbreaks with low mortality (influenza), moderate contagious outbreaks with potentially high morbidity, but for which treatment is available (TB) and the rate of tertiary spread is not rapid. Only one study described an outbreak of COVID-19 in prison.^70^ The applicability of this evidence base to COVID-19 is not clear as it is highly contagious (less than measles and varicella, but more than others), with moderate morbidity but very rapid spread (typically more than other infections). Importantly, unlike all the other contagious diseases reviewed, there is no known appreciable population immunity to COVID-19, permitting its rapid propagation through susceptible populations due to absence of any herd immunity. As such, an uncontrolled COVID-19 outbreak in a confined setting such as a prison would be expected to lead large numbers within a few days, with the potential to completely overwhelm the institution rapidly. As the COVID-19 pandemic proceeds, there is an urgent need for extensive interagency collaboration. Information sharing between institutions and public health authorities regarding measures employed and indications of effectiveness could help improve preparedness for future prison outbreaks.

### Challenges

Six main challenges to managing outbreaks of contagious infections were identified, with relevance to different interventions (table 4). Low uptake of interventions, withholding of symptoms, limited capacity of staff and the physical environment were shown to reduce the impact of specific interventions in included studies. More broadly, the identified lack of prison-specific guidelines and the prioritisation of security needs, which are clear implications from some reviewed papers but not specifically recommended, may have an important bearing on all aspects of outbreak management. Other challenges include the efficacy of screening for the acute disease and for immunity. Screening for acute disease is moderately useful for TB, and not shown to be useful for influenza. Screening of immune status has been shown to be effective for measles and mumps. The time to discover the outbreak before onward transmission (interval time) has occurred is another challenge. Finally, there is a lack of effective interventions for some contagious diseases—immunisation to interrupt transmission is potentially useful for influenza but not for TB, whereas the role of antivirals and antibacterial agents for prophylaxis is unproven. Maintaining independent investigations of deaths and continuing official complaints procedures, which are important to learn lessons and to improve PIP-staff relationships, needs consideration. Further, any reduction in peer to peer support within prison, including for suicidal PIP, should be addressed, especially with risks to mental health from measures such as isolation. It is also important to ensure that vulnerable PIP are not released to environments where infection is prevalent, such as to a home where a family member is symptomatic, or conversely to expose any vulnerable persons in the community to a released PIP who is infectious.

**Table 4:** Implications of challenges to managing outbreaks of contagious infections in prisons on potential interventions

	Immunisation	Restrictions, isolation and quarantine	Screening	Contact tracing	Epidemiological surveillance
Low uptake of interventions^49 55^	x				
PIP withholding early symptoms to avoid restrictions^44 51 70^		x	x		
Limits of staff capacity^55^	x		x	x	x
Limits of physical environment^51 58^		x			
Lack of prison specific guidelines^45 47 48 50 51 53 56 60 63 66^	x	x	x	x	x
Prioritisation of security over health needs^50^	x	x	x	x	x

PIP, people in prison.

### Research gaps

The most notable gap was how to most effectively reduce the prison population during any outbreak. This would involve releasing persons at low risk of future serious offending and not incarcerating people charged with low-level offences.^80 81^ For example, in some jurisdictions, judges have been asked to consider the impact of COVID-19 on prison conditions when making decisions on custodial sentences.^82^ Lean, efficient and transparent methods are required to assist if they are to garner public confidence.^83^ Some jurisdictions have released individuals purely on the basis that they are close to their release date, while others have included low-risk PIP, such as pregnant women and older adults.^84^ Whatever approaches are used, a wider discussion is required on how to balance public safety with public health, which may include economic modelling,^85^ and ethical and legal considerations.^86 87^ Other gaps were in relation to protective equipment—only one study recommended all individuals entering AII rooms wear special masks.^57^ This underscores another major gap—that the health and well-being of prison staff has not been sufficiently considered in previous work. This will include having adequate levels of staffing, who have access to updated information on infection risks, and training that underlines the importance of strong links with public health who can test, trace and isolate prison officers and healthcare staff. Descriptions of environmental conditions prior to the outbreak represents an additional research gap. This is necessary to evaluate the susceptibility and preparedness of correctional facilities to handle future outbreaks, and examine the impact of interventions postoutbreak. Areas that could be surveyed include the extent of overcrowding, ventilation, sanitation and hygiene, pre-existing access to healthcare services and screening capacities for such diseases.^1 3^ Included studies did not address the potential harms associated with prolonged infection control strategies, such as isolation and physical distancing.^88^ This may particularly affect the mental health of PIP, who have higher background of mental illness than community peers^89^ and the detrimental effects of solitary confinement in correctional facilities on mental health need to be carefully weighed up,^90^ including potentially elevating risk of self-harm. Therefore, mental health should be at the forefront of considerations when implementing infection control measures in prisons to mitigate adverse consequences for PIP.^91–93^ Finally, a key consideration that is not addressed in any of the included papers are the consequences of how health services in prison are commissioned and their level of integration with community health systems. Separate systems are more prone to breakdowns and delays in follow-up care, which will be necessary to ensure treatment completion and epidemiological surveillance.

### Limitations

All included studies were based in high-income countries and some recommendations may not translate to low-income and middle-income settings. There is a pressing need for research to be conducted in those settings, who make up a majority of PIP worldwide.^94^ Prisons in these countries will be different, and the translatability of the review findings needs to be investigated. Although we did not include a formal quality assessment, all included studies were observational and we did not identify any trials.

## Conclusion

Although there is some observational evidence on highly contagious disease outbreaks in prison, COVID-19 represents a unique challenge to prisons due to its distinct epidemiology. Previous outbreaks of other diseases have been self-limiting, while prisons could become reservoirs for COVID-19 infection to the community if appropriate public health measures are not instituted. All prisons should consider communicating clear and regular health information updates to PIP and staff, isolating all new prison entrants, contact tracing, and providing a highly responsive testing regime to all people who are incarcerated and prison staff, including prioritisation for early adoption and implementation of diagnostic assays and tests. Overcrowding needs to be tackled by reducing the number of new arrivals and releasing low risk persons, while taking into account that many individuals released from prisons will find themselves in shelters and other unstable housing situations, which necessitates planning and coordination between public agencies. Such planning will require time and interagency cooperation so that risks are mitigated, including of initial placements breaking down.
