# Supplementary material for: Managing outbreaks of highly contagious diseases in prisons: a systematic review
Source: BMJ Glob Health. 2020 Nov 16;5(11):e003201. doi: 10.1136/bmjgh-2020-003201 (PMC7670855; doi:10.1136/bmjgh-2020-003201)
Supplement: Supplementary data [file bmjgh-2020-003201supp001.pdf]

BMJ Publishing Group Limited (BMJ) disclaims all liability and responsibility arising from any reliance placed on this supplemental material which has been supplied by the author(s)

Managing outbreaks of highly contagious diseases in prisons

Supplementary appendix A
Appendix 1: Search strategy and results
Appendix 2: PRISMA checklist

Beaudry G, *et al. BMJ Global Health* 2020; 5:e003201. doi: 10.1136/bmjgh-2020-003201

Managing outbreaks of highly contagious diseases in prisons

APPENDIX 1: SEARCH STRATEGY

*The search strategies were run on March 26, 2020 by Elinor Harriss, librarian at the Bodleian Health Care Libraries, University of Oxford.

Medline (Ovid MEDLINE® Epub Ahead of Print, In-Process & Other Non-Indexed Citations, Ovid MEDLINE® Daily and Ovid MEDLINE®) 1946 to present

----

1 Prisons/ (9485)
2 Prisoners/ (16341)
3 (prison* or incarcerat* or custod* or imprison* or detain* or inmate* or jail* or gaol* or penal or penitentiary or "correctional facilit*" or probation*).ti,ab. (37536)
4 1 or 2 or 3 (45181)
5 disease outbreaks/ or epidemics/ or pandemics/ or space-time clustering/ (93455)
6 "out-break*".ti,ab. (126)
7 (outbreak* or transmission or epidemic* or pandemic*).ti,ab. (520426)
8 5 or 6 or 7 (545861)
9 4 and 8 (2144)

Embase 1974 to present

----

1 prison/ (14614)
2 prisoner/ (16087)
3 (prison* or incarcerat* or custod* or imprison* or detain* or inmate* or jail* or gaol* or penal or penitentiary or "correctional facilit*" or probation*).ti,ab. (45363)
4 1 or 2 or 3 (52663)
5 epidemic/ or pandemic/ or pandemic influenza/ (114304)
6 "out-break*".ti,ab. (210)
7 (outbreak* or transmission or epidemic* or pandemic*).ti,ab. (574527)
8 5 or 6 or 7 (603978)
9 4 and 8 (2581)

PsycINFO 1806 to present

----

1 prisons/ (7074)
2 prisoners/ (10559)
3 (prison* or incarcerat* or custod* or imprison* or detain* or inmate* or jail* or gaol* or penal or penitentiary or "correctional facilit*" or probation*).ti,ab. (49649)
4 1 or 2 or 3 (50469)
5 exp epidemics/ (3332)
6 "out-break*".ti,ab. (18)
7 (outbreak* or transmission or epidemic* or pandemic*).ti,ab. (52907)
8 5 or 6 or 7 (53152)
9 4 and 8 (675)

Global Health <1973 to 2020 Week 11>

----

1 correctional institutions/ (2253)
2 prisoners/ (3035)
3 (prison* or incarcerat* or custod* or imprison* or detain* or inmate* or jail* or gaol* or penal or penitentiary or "correctional facilit*" or probation*).ti,ab. (6517)
4 1 or 2 or 3 (6707)
5 outbreaks/ or exp epidemics/ (52687)
6 "out-break*".ti,ab. (71)
7 (outbreak* or transmission or epidemic* or pandemic*).ti,ab. (202331)
8 5 or 6 or 7 (204045)
9 4 and 8 (1260)

Cochrane Database of Systematic Reviews
Issue 3 of 12, March 2020

Managing outbreaks of highly contagious diseases in prisons

Cochrane Central Register of Controlled Trials
Issue 3 of 12, March 2020

#1 MeSH descriptor: [Prisons] explode all trees 117
#2 MeSH descriptor: [Prisons] explode all trees 117
#3 prison* or incarcerat* or custod* or imprison* or detain* or inmate* or jail* or gaol* or penal or penitentiary or "correctional facilit*" or probation* 2582
#4 #1 or #2 or #3 2583
#5 MeSH descriptor: [Disease Outbreaks] this term only 192
#6 MeSH descriptor: [Epidemics] this term only 20
#7 MeSH descriptor: [Pandemics] explode all trees 49
#8 "out-break*" 4
#9 outbreak* or transmission or epidemic* or pandemic* 14074
#10 #5 or #6 or #7 or #8 or #9 14077
#11 #4 and #10 117

(37 Cochrane Reviews; 74 Trials)

Web of Science Core Collection
TOPIC: (prison* or incarcerat* or custod* or imprison* or detain* or inmate* or jail* or gaol* or penal or penitentiary or "correctional facilit*" or probation*) AND TOPIC: ("out-break*" or outbreak* or transmission or epidemic* or pandemic*)

ProQuest National Criminal Justice Reference Service (NCJRS) Abstracts Database

(ti("out-break*" OR outbreak* OR transmission OR epidemic* OR pandemic*) OR ab("out-break*" OR outbreak* OR transmission OR epidemic* OR pandemic*)) AND (ti(prison* OR incarcerat* OR custod* OR imprison* OR detain* OR inmate* OR jail* OR gaol* OR penal OR penitentiary OR "correctional facilit*" OR probation*) OR ab(prison* OR incarcerat* OR custod* OR imprison* OR detain* OR inmate* OR jail* OR gaol* OR penal OR penitentiary OR "correctional facilit*" OR probation*))

Search Results

	Search results 26/03/2020	Search results 28/07/2020
Ovid Medline	2144	2239
Ovid Embase	2581	2687
Ovid PsycINFO	675	695
Ovid Global Health	1260	1283
Cochrane Database of Systematic Reviews and Cochrane CENTRAL	111	822 (186 Cochrane Reviews; 636 Trials)
Web of Science Core Collection	2470	2568
ProQuest NCJRS	541	543
TOTAL	9782	10837
Total after deduplication	5071	**5959**
Unique since 26/03/2020		**912**

Managing outbreaks of highly contagious diseases in prisons

APPENDIX 2: PRISMA CHECKLIST

Section/topic	#	Checklist item	Reported on page #
**TITLE**			
Title	1	Identify the report as a systematic review, meta-analysis, or both.	1
**ABSTRACT**			
Structured summary	2	Provide a structured summary including, as applicable: background; objectives; data sources; study eligibility criteria, participants, and interventions; study appraisal and synthesis methods; results; limitations; conclusions and implications of key findings; systematic review registration number.	2
**INTRODUCTION**			
Rationale	3	Describe the rationale for the review in the context of what is already known.	4-5
Objectives	4	Provide an explicit statement of questions being addressed with reference to participants, interventions, comparisons, outcomes, and study design (PICOS).	5
**METHODS**			
Protocol and registration	5	Indicate if a review protocol exists, if and where it can be accessed (e.g., Web address), and, if available, provide registration information including registration number.	5
Eligibility criteria	6	Specify study characteristics (e.g., PICOS, length of follow-up) and report characteristics (e.g., years considered, language, publication status) used as criteria for eligibility, giving rationale.	5
Information sources	7	Describe all information sources (e.g., databases with dates of coverage, contact with study authors to identify additional studies) in the search and date last searched.	5
Search	8	Present full electronic search strategy for at least one database, including any limits used, such that it could be repeated.	2-3 (appendix A)
Study selection	9	State the process for selecting studies (i.e., screening, eligibility, included in systematic review, and, if applicable, included in the meta-analysis).	4-5
Data collection process	10	Describe method of data extraction from reports (e.g., piloted forms, independently, in duplicate) and any processes for obtaining and confirming data from investigators.	5-6
Data items	11	List and define all variables for which data were sought (e.g., PICOS, funding sources) and any assumptions and simplifications made.	6

Managing outbreaks of highly contagious diseases in prisons

Risk of bias in individual studies	12	Describe methods used for assessing risk of bias of individual studies (including specification of whether this was done at the study or outcome level), and how this information is to be used in any data synthesis.	6
Summary measures	13	State the principal summary measures (e.g., risk ratio, difference in means).	6
Synthesis of results	14	Describe the methods of handling data and combining results of studies, if done, including measures of consistency (e.g., I^2) for each meta-analysis.	6
Risk of bias across studies	15	Specify any assessment of risk of bias that may affect the cumulative evidence (e.g., publication bias, selective reporting within studies).	NA
Additional analyses	16	Describe methods of additional analyses (e.g., sensitivity or subgroup analyses, meta-regression), if done, indicating which were pre-specified.	NA
**RESULTS**			
Study selection	17	Give numbers of studies screened, assessed for eligibility, and included in the review, with reasons for exclusions at each stage, ideally with a flow diagram.	7
Study characteristics	18	For each study, present characteristics for which data were extracted (e.g., study size, PICOS, follow-up period) and provide the citations.	7-11
Risk of bias within studies	19	Present data on risk of bias of each study and, if available, any outcome level assessment (see item 12).	NA
Results of individual studies	20	For all outcomes considered (benefits or harms), present, for each study: (a) simple summary data for each intervention group (b) effect estimates and confidence intervals, ideally with a forest plot.	NA
Synthesis of results	21	Present results of each meta-analysis done, including confidence intervals and measures of consistency.	12-18
Risk of bias across studies	22	Present results of any assessment of risk of bias across studies (see Item 15).	NA
Additional analysis	23	Give results of additional analyses, if done (e.g., sensitivity or subgroup analyses, meta-regression [see Item 16]).	NA
**DISCUSSION**			
Summary of evidence	24	Summarize the main findings including the strength of evidence for each main outcome; consider their relevance to key groups (e.g., healthcare providers, users, and policy makers).	18-20
Limitations	25	Discuss limitations at study and outcome level (e.g., risk of bias), and at review-level (e.g., incomplete retrieval of identified research, reporting bias).	22
Conclusions	26	Provide a general interpretation of the results in the context of other evidence, and implications for future research.	22-23
**FUNDING**			
Funding	27	Describe sources of funding for the systematic review and other support (e.g., supply of data); role of funders for the systematic review.	29

From: Moher D, Liberati A, Tetzlaff J, Altman DG, The PRISMA Group (2009). Preferred Reporting Items for Systematic Reviews and Meta-Analyses: The PRISMA Statement. PLoS Med 6(7): e1000097. doi:10.1371/journal.pmed1000097

For more information, visit: **www.prisma-statement.org**.
